# Antimicrobial Resistance at the Human–Animal–Environment Interface: A One Health Perspective on Drivers, Transmission, and Public Health Responses

**DOI:** 10.1002/hsr2.72964

**Published:** 2026-08-04

**Authors:** Nurshad Ali

**Affiliations:** ^1^ Department of Biochemistry and Molecular Biology Shahjalal University of Science and Technology Sylhet Bangladesh

**Keywords:** antimicrobial resistance, human–animal–environment interface, One Health, stewardship, surveillance

## Abstract

**Background:**

Antimicrobial resistance (AMR) is a global health threat driven by interconnected antimicrobial use and environmental pressures across human, animal, and ecological systems. This review synthesizes current evidence on the major drivers, transmission pathways, reservoirs, surveillance challenges, and public health responses associated with AMR at the human–animal–environment interface.

**Methods:**

Relevant literature published up to March 2026 was identified through searches of PubMed, Scopus, Web of Science, and Google Scholar using terms related to AMR, One Health, antimicrobial use, transmission, surveillance, and public health responses. Peer‐reviewed studies, systematic reviews, and reliable reports were considered, while duplicate, unrelated, non‐English, and non‐peer‐reviewed publications were excluded.

**Results:**

AMR is driven by inappropriate antimicrobial use in human healthcare, livestock and aquaculture production, and environmental contamination from wastewater, agricultural runoff, and pharmaceutical discharges. Resistant organisms and antimicrobial resistance genes circulate across sectors through food, water, direct contact, occupational exposure, wildlife, and horizontal gene transfer. Major gaps remain in integrated surveillance, cross‐sector data sharing, diagnostic capacity, laboratory infrastructure, and implementation, particularly in resource‐limited settings. These challenges are compounded by fragmented governance, inadequate regulation, limited stewardship, and poor coordination among human, animal, and environmental health sectors, hindering effective AMR prevention and control.

**Conclusions:**

Containing AMR requires coordinated One Health action integrating antimicrobial stewardship, infection prevention, improved diagnostics, harmonized surveillance, environmental monitoring, and cross‐sector data sharing. Strengthening implementation capacity and equitable collaboration is essential for effective and sustainable AMR control.

## 1 Introduction

Antimicrobial resistance (AMR) has become one of the biggest public health threats of the 21st century. It undermines years of progress in preventing and treating infectious diseases. Resistant bacterial infections lead to more illness and death, longer hospital stays, and substantial economic costs, putting significant pressure on health systems everywhere [1]. Recent estimates show that bacterial AMR directly caused about 1.27 million deaths in 2019 and contributed to nearly 5 million deaths overall. This burden falls most heavily on low‐ and middle‐income countries (LMICs). If no effective action is taken, the global burden of bacterial AMR is projected to increase substantially, with major consequences for mortality and health systems by 2050 [2].

AMR emerges and spreads due to complex factors that go beyond just human health settings. The misuse and overuse of antimicrobials in medicine, livestock production, aquaculture, and agriculture have sped up the selection of resistant microorganisms [3]. These resistant bacteria and resistance genes move across different sectors through direct contact, the food chain, water systems, and environmental contamination. This shows how interconnected AMR really is [4, 5]. Environmental sources, like wastewater, surface water, soil, and sediments, play a crucial role in maintaining and spreading antimicrobial‐resistant bacteria and resistance genes, but they are often overlooked in many monitoring and control plans [6, 7].

Given this complexity, the One Health approach has become an important way to address AMR. One Health highlights the links between human health, animal health, and environmental health. It calls for coordinated actions across different sectors and disciplines [8]. International organizations, including the World Health Organization (WHO), the Food and Agriculture Organization (FAO), and the World Organization for Animal Health (WOAH), have supported One Health as a key part of global AMR action plans. However, even with increasing political commitment, implementation is uneven. There are still gaps in integrated monitoring, data sharing, and policy coordination, especially in resource‐limited areas [9].

The aim of this review is to synthesize current evidence on AMR at the intersection of human, animal, and environmental health using a One Health perspective. Specifically, the review aims to (i) summarize the main drivers and sources of AMR in human, animal, and environmental sectors; (ii) describe key transmission pathways that connect these areas; and (iii) examine existing monitoring systems, public health measures, and policy responses related to One Health AMR control. By providing a cohesive overview, this review hopes to inform public health practitioners, policymakers, and researchers while highlighting key areas for coordinated action to reduce the global threat of AMR.

A comprehensive literature search was conducted in PubMed, Scopus, Web of Science, and Google Scholar to identify relevant studies published up to March 2026. A combination of terms related to AMR, One Health, human–animal–environment interfaces, transmission, drivers, surveillance, and public health responses was used. Peer‐reviewed articles, systematic reviews, and reliable reports were included, whereas duplicate, non‐English, unrelated, and non‐peer‐reviewed publications were excluded.

## 2 Conceptual Framework: One Health and Antimicrobial Resistance

One Health integrates human, animal, and environmental health to explain how resistant microorganisms and resistance genes develop, persist, and spread through connected biological and ecological systems [10].

AMR is not limited to human clinical settings. It is influenced by linked practices and exposures in medical, veterinary, agricultural, and environmental fields. In human health, inappropriate antimicrobial prescribing, poor infection prevention and control, and limited access to diagnostics create conditions that favor resistant pathogens. Meanwhile, the extensive use of antimicrobials for preventing disease, promoting growth, and treating illnesses in food‐producing animals and aquaculture largely drives resistance selection in bacteria associated with animals [11]. These resistant organisms can be passed to humans through direct contact, workplace exposure, or consuming contaminated food.

The environment acts as both a reservoir and a pathway for AMR. Antimicrobials, resistant bacteria, and resistance genes enter the environment through wastewater, pharmaceutical discharge, agricultural runoff, and improper medical waste disposal. Aquatic and land environments help resistant genes persist and spread among various microbial communities. This process increases and redistributes resistance across sectors [6, 7]. This ecological aspect highlights the environment as an active player in the AMR cycle rather than simply a passive receiver.

AMR transmission can be seen as a dynamic network connecting humans, animals, and the environment through various two‐way pathways. These pathways include the food chain, water systems, soil contamination, wildlife, and human movement. Horizontal gene transfer (HGT) methods, such as conjugation, transformation, and transduction, allow resistance genes to move between bacterial species and ecological niches, making containment efforts even more challenging [4, 5]. A One Health model thus presents AMR as a broader systems issue that needs integrated monitoring, aligned policies, and cooperative actions across sectors to effectively lessen its impact on public health.

## 3 Antimicrobial Resistance in Human Health

AMR is a serious threat to human health. It undermines the effective treatment of common bacterial infections and raises the risk of severe disease outcomes. Several resistant pathogens are especially concerning due to their worldwide presence, clinical impact, and limited treatment options (Table 1). The WHO has prioritized pathogens such as carbapenem‐resistant *Acinetobacter baumannii*, *Pseudomonas aeruginosa*, and Enterobacterales, as well as methicillin‐resistant *Staphylococcus aureus* (MRSA), vancomycin‐resistant *Enterococcus* spp. (VRE), and drug‐resistant *Mycobacterium tuberculosis* [12]. These organisms account for a significant number of healthcare‐associated and community‐acquired infections globally. They also contribute greatly to morbidity and mortality to AMR [1].

Clinical factors play a key role in the rise and spread of AMR. Inappropriate antibiotic prescribing, such as treating viral infections unnecessarily, using broad‐spectrum agents too much, and incorrect dosing or treatment duration, is still common [13]. Limited diagnostic capabilities and poor management of antibiotics, especially in LMICs, make the issue worse. Healthcare settings contribute to the selection and spread of resistant pathogens due to high antibiotic use, invasive procedures, long hospital stays, and weak infection prevention and control [14]. As a result, hospitals and long‐term care facilities can become major sources of multidrug‐resistant organisms and may spread these to the wider community.

AMR is also spreading more in communities through close human contact, contaminated food and water, poor sanitation, and environmental factors. Community‐associated MRSA, ESBL‐producing *Escherichia coli*, and fluoroquinolone‐resistant *Salmonella* spp. illustrate resistant pathogens found outside healthcare settings [15]. International travel, movement of people, and crowded urban areas help this spread across regions. Tackling AMR requires coordinated surveillance and actions that connect clinical, community, and environmental settings within a broader One Health approach.

In many LMICs, community transmission of resistant pathogens is worsened by social and structural factors. These include the easy availability of antibiotics without prescriptions, self‐medication, informal healthcare providers, and patients' expectations for antibiotic prescriptions. Poor water, sanitation, and hygiene systems help keep resistant organisms circulating outside healthcare settings. This situation highlights the need for community‐focused interventions to address AMR [2, 16].

## 4 Antimicrobial Resistance in Animal Health and Food Production

Using antimicrobials in animal health and food production is crucial in the global rise and spread of AMR. Antimicrobials are commonly given to livestock, poultry, and fish for treating illnesses, preventing diseases, and in some cases, promoting growth. While these practices have helped improve animal productivity, their widespread and often careless use puts significant pressure on bacterial populations, leading to the development of resistant strains that can affect both animal and human health [11].

Worldwide, food‐producing animals make up a large share of total antimicrobial use, with especially fast increases seen in intensively farmed poultry and aquaculture. In aquaculture systems, antimicrobial use in open environments can promote resistant bacteria in farmed animals and surrounding ecosystems, increasing the potential for resistance‐gene dissemination [17]. In land‐based livestock systems, high animal density and routine use of prophylactic and metaphylactic antimicrobials add to the growth and persistence of resistant bacteria in herds and flocks.

The rise of antimicrobial‐resistant bacteria in animals directly impacts the food chain. Resistant pathogens and commensal bacteria can contaminate meat, milk, eggs, and other animal products during slaughter, processing, and handling. Notable examples include extended‐spectrum β‐lactamase (ESBL)‐producing *Escherichia coli*, fluoroquinolone‐resistant *Campylobacter* spp., and multidrug‐resistant *Salmonella* spp. These have often been found in food products and linked to human infections [18]. Once these bacteria enter human populations, they can cause infections that are harder to treat and can also pass on resistance genes to other bacteria that infect humans.

Besides foodborne transmission, occupational exposure is a significant but often overlooked route for the spread of AMR. Farmers, veterinarians, slaughterhouse workers, and others involved in animal care and food processing face a higher risk of colonization or infection with resistant bacteria through direct contact with animals, animal waste, and contaminated areas [19]. These individuals can act as bridges for spreading resistant organisms between animal environments and communities. These factors highlight the need for combining antimicrobial stewardship, biosecurity, and monitoring in animal production systems as part of a broad One Health approach to lessen the public health impact of AMR.

Evidence from South and Southeast Asia shows that intensive poultry, livestock, and aquaculture systems can amplify AMR through high stocking densities, limited veterinary oversight, and non‐therapeutic antimicrobial use [20, 21]. Resistant bacteria may spread to humans through contaminated food, farm environments, and direct contact. High levels of multidrug‐resistant *Escherichia coli*, *Salmonella*, and *Campylobacter*, alongside evidence of animal–human bacterial overlap, highlight food production systems as key points in the One Health AMR continuum contact [11, 21, 22].

## 5 Environmental Dimensions of Antimicrobial Resistance

The environment plays a key role in the rise, persistence, and spread of AMR. It serves as both a source and a pathway for resistant bacteria and antimicrobial resistance genes (ARGs). Aquatic and terrestrial environments, including surface water, groundwater, soil, and wastewater systems, continuously receive antimicrobials, resistant microorganisms, and genes from human, animal, and industrial sources [23]. These inputs create conditions that promote the selection and spread of resistance [6]. Environmental compartments, including water and soil, contain clinically important resistant organisms like carbapenem‐resistant *Acinetobacter baumannii* and *Klebsiella pneumoniae*. These organisms help spread resistance across different sectors (Table 1).

Wastewater systems are important environmental reservoirs and pathways for AMR because treatment processes may not fully remove antimicrobial residues, resistant bacteria, or ARGs, allowing them to enter surface and coastal waters [24, 25]. Similarly, soils that receive untreated or poorly treated manure and sewage sludge build up antimicrobial residues and resistant bacteria, leading to long‐term persistence of ARGs in agricultural ecosystems.

Pharmaceutical waste and agricultural runoff are major causes of environmental AMR contamination. Wastewater from pharmaceutical manufacturing sites often contains very high levels of antibiotics, far surpassing therapeutic amounts. This creates intense selective pressure on microbial communities in the environment [26]. In agricultural areas, runoff from livestock farms and aquaculture operations adds antimicrobials and resistant bacteria to nearby waterways and sediments. These contributions encourage resistance growth not just in harmful bacteria but also in environmental commensals, which can transfer ARGs to human and animal pathogens [27].

The spread of resistance genes is made worse by HGT methods, including conjugation, transformation, and transduction. Aquatic and soil environments provide ideal conditions for microbial interactions and gene exchange, allowing ARGs to transfer between bacterial species and ecological niches [7, 28]. Mobile genetic elements, such as plasmids, integrons, and transposons, aid this process and speed up the spread of resistance among environmental, animal, and human‐associated bacteria [29]. The environmental resistome therefore links human antimicrobial use with clinical AMR outcomes.

Environmental spread of AMR may worsen due to climate change. Flooding, rising temperatures, and changing water cycles can help resistant bacteria and resistance genes move and survive across water systems, soils, and agricultural areas [30, 31].

## 6 Transmission Pathways at the Human, Animal, and Environment Interface

**FIGURE 1:**
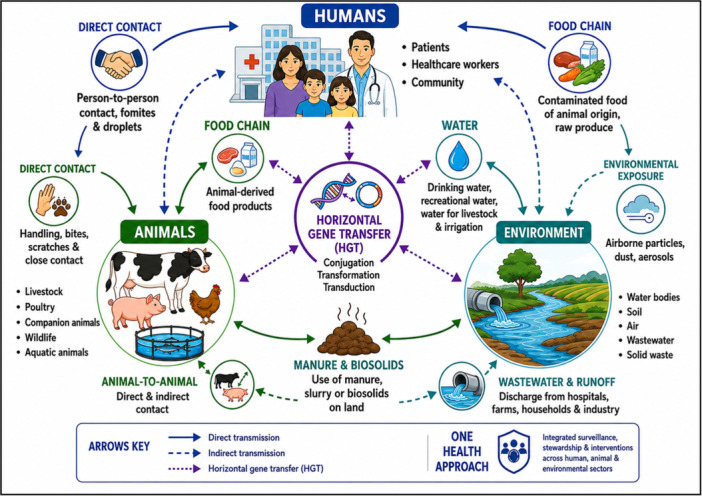
Transmission pathways, reservoirs, and dissemination of antimicrobial resistance across the human–animal–environment interface within the One Health framework. Antimicrobial resistance (AMR) spreads in both directions among humans, animals, and the environment through direct contact, the food chain, water, wastewater, manure, biosolids, and environmental exposure. Horizontal gene transfer (HGT) through conjugation, transformation, and transduction helps spread antimicrobial resistance genes (ARGs) within and between these connected sectors. The One Health approach emphasizes integrated surveillance, responsible antimicrobial use, antimicrobial stewardship, and coordinated efforts to reduce AMR spread across human, animal, and environmental systems.

Table 2 and Figure 1 outline the main drivers, transmission routes, and key interventions across human, animal, and environmental sectors. It highlights important points for One Health action. AMR transmission occurs through interconnected direct and indirect pathways linking humans, animals, and the environment.

**TABLE 1:** Priority antimicrobial‐resistant pathogens, associated resistance mechanisms, and their relevance across human, animal, and environmental sectors.

Pathogen/organism	Resistance mechanism(s)	Sectoral relevance	Examples of transmission	References
*Escherichia coli* (ESBL‐producing)	β‐lactamase enzymes; plasmid‐mediated resistance	Humans, livestock, environment	Contaminated food, water, occupational exposure	[6, 18]
*Salmonella* spp. (multidrug‐resistant)	Efflux pumps, target mutations	Humans, poultry, livestock	Foodborne transmission, water, soil	[11]
*Staphylococcus aureus* (MRSA)	mecA gene, penicillin‐binding protein alteration	Humans, livestock	Direct contact, healthcare settings, farm environments	[4]
*Enterococcus* spp. (VRE)	VanA/VanB gene clusters	Humans, animals	Healthcare‐associated, foodborne, environmental	[1]
*Pseudomonas aeruginosa* (multidrug‐resistant)	Efflux pumps, β‐lactamases, porin loss	Humans, water systems	Hospitals, contaminated water, soil	[32, 33]
*Campylobacter* spp. (fluoroquinolone‐resistant)	Target‐site mutations in gyrA (particularly T86I); efflux pumps such as CmeABC	Humans, poultry	Foodborne transmission, direct animal contact, and environmental contamination	[15, 34]
*Acinetobacter baumannii* (carbapenem‐resistant)	β‐lactamases, efflux pumps	Humans, environmental reservoirs	Healthcare settings, wastewater	[4]
*Klebsiella pneumoniae* (ESBL and carbapenem‐resistant)	β‐lactamases, carbapenemases	Humans, animals, environment	Food, water, healthcare settings	[1]

Abbreviations: CmeABC, *Campylobacter* multidrug efflux complex; DNA gyrase subunit A gene; ESBL, extended‐spectrum β‐lactamase; gyrA, mecA, methicillin resistance gene A; MDR, multidrug‐resistant; MRSA, methicillin‐resistant *Staphylococcus aureus*; T86I, threonine‐to‐isoleucine substitution at position 86; VanA/VanB, vancomycin‐resistance gene clusters A and B; VRE, vancomycin‐resistant *Enterococcus*.

**TABLE 2:** Summary of antimicrobial resistance drivers, transmission pathways, and interventions across human, animal, and environmental sectors.

Sector	Key AMR drivers	Major transmission pathways	Public health interventions	References
Human health	‐ Inappropriate prescribing, overuse, self‐medication‐ Broad‐spectrum antibiotic use‐ Inadequate IPC‐ Limited diagnostics and stewardship programs	‐ Direct person‐to‐person transmission‐ Healthcare‐associated infections‐ Contaminated water & food	‐ Antimicrobial AMS‐ Strengthened IPC in hospitals & communities‐ Public education and behavior change	[1, 4, 13]
Animal health and food production	‐ Therapeutic, prophylactic, and growth‐promoting antimicrobial use in livestock, poultry, and aquaculture‐ High‐density farming practices‐ Poor biosecurity	‐ Food chain contamination (meat, milk, eggs, and seafood)‐ Direct contact with farm animals (occupational exposure)‐ Runoff into soil and water	‐ Veterinary AMS‐ Biosecurity & vaccination‐ Prudent use regulations & alternatives to antibiotics	[11, 18, 19]
Environment	‐ Wastewater from humans, farms, and pharmaceutical industries‐ Agricultural runoff & manure application‐ Persistence of antimicrobials and resistant bacteria in soil & water	‐ Contaminated surface and groundwater‐ Soil and sediment reservoirs‐ Wildlife vectors and ecosystem spread	‐ Environmental monitoring & surveillance‐ Wastewater treatment and pollution control‐ Regulations for pharmaceutical discharge	[6, 25, 28]

*Note:* Summarizes key factors that promote AMR in humans, animals, and the environment. It outlines the main ways pathogens and genes spread. It also presents sector‐specific strategies to reduce the issue. The text emphasizes the need for coordinated One Health actions.

Abbreviations: AMR, antimicrobial resistance; AMS, antimicrobial stewardship; HGT, horizontal gene transfer; IPC, infection prevention and control; LMICs, low‐ and middle‐income countries.

Direct transmission happens through physical contact between humans and animals or through contaminated environments. Occupational exposure among farmers, veterinarians, slaughterhouse workers, and food handlers is a well‐documented route for transferring resistant bacteria from animals to humans [19, 35]. Similarly, person‐to‐person transmission in households, healthcare facilities, and communities spreads resistant pathogens like MRSA and multidrug‐resistant Enterobacterales. Indirect transmission pathways involve intermediate vehicles such as food, water, soil, and surfaces, which allow resistant organisms to spread across populations and geographic areas without direct contact [36].

The food chain is a key route for AMR transmission from animals to humans. Resistant bacteria from livestock, poultry, and aquaculture can contaminate meat, milk, eggs, and seafood during production, processing, and distribution. Consuming or handling contaminated food products has been linked to human infections caused by ESBL‐producing *Escherichia coli*, multidrug‐resistant *Salmonella* spp., and fluoroquinolone‐resistant *Campylobacter* spp [18]. Global food trade increases these risks by enabling rapid movement of resistant strains across borders.

Water systems are another critical route for spreading AMR. Municipal wastewater treatment plants receive waste containing antibiotics, resistant bacteria, and ARGs from households, hospitals, farms, and pharmaceutical industries [37]. Incomplete removal during treatment allows these contaminants to enter surface waters and irrigation systems, leading to human exposure through drinking water, recreational activities, and crop irrigation [24, 25]. Aquatic ecosystems also serve as mixing zones where bacteria from humans, animals, and the environment interact, increasing chances for resistance gene exchange.

Ecosystems and wildlife play a growing role in AMR transmission. Wildlife exposed to contaminated environments can acquire resistant bacteria and transport them across ecosystems and human–animal boundaries [38]. Soil and sediment enriched with antimicrobial residues from agricultural runoff or sewage sludge create long‐term reservoirs for resistant bacteria. This sustains environmental resistomes that can re‐enter human and animal populations [6].

HGT is a key method that allows AMR to spread across different sectors. Through conjugation, transformation, and transduction, ARGs on mobile genetic elements such as plasmids, integrons, and transposons can move between different bacterial species and genera. Environmental and gut microbiomes provide ideal conditions for these exchanges, allowing resistance traits selected in one sector, like agriculture, to show up in clinically relevant human pathogens [4, 28]. This genetic connectivity highlights AMR as a systems‐level issue requiring coordinated One Health surveillance and interventions.

## 7 Mechanisms and Major Drivers of Antimicrobial Resistance

AMR arises from a mix of biological processes and human activities that speed up the rise, selection, and spread of resistant microorganisms [39]. At the microbial level, resistance mechanisms include enzymatic drug inactivation, changes to antimicrobial targets, lower membrane permeability, and active removal of antimicrobial agents. Examples include β‐lactamase production in Enterobacterales, target site mutations that cause fluoroquinolone resistance, and overexpression of efflux pumps in *Pseudomonas aeruginosa* [32, 40]. These mechanisms help bacteria survive exposure to antimicrobials and endure selective pressure.

Genetic factors responsible for resistance are often found on mobile genetic elements like plasmids, integrons, and transposons [41]. These elements enable HGT through conjugation, transformation, and transduction. HGT allows resistance genes to move quickly between bacterial species and across ecological areas. This links resistance that emerges in animals or the environment to important human pathogens [42]. This genetic movement is a major reason for the fast global spread of AMR.

Human activities serve as major drivers that intensify these biological processes. Inappropriate antimicrobial use in human medicine—including overprescribing, self‐medication, incomplete treatment courses, and extensive reliance on broad‐spectrum antibiotics—creates selective pressure that promotes the emergence and spread of resistance [43, 44]. Limited diagnostic capabilities and weak antimicrobial stewardship programs worsen the issue, especially in LMICs [2, 45]. Healthcare settings with high antimicrobial use and poor infection control practices become hotspots for the emergence and spread of multidrug‐resistant organisms.

Outside of clinical settings, use of antimicrobials in livestock, poultry, and aquaculture also plays a big role in AMR development. Antimicrobials used for disease prevention and growth promotion create selective pressure on animal‐associated bacteria, leading to resistant strains that can spread through the food chain and the environment [11]. Environmental factors add to the problem. Waste from pharmaceutical manufacturing, agricultural runoff, and wastewater discharge introduce antimicrobials and resistant bacteria into natural ecosystems, promoting resistance selection and gene transfer among various microbial communities [6].

Socioeconomic factors like population growth, urbanization, global travel, and trade also help spread AMR by increasing connections between human, animal, and environmental systems [4]. Together, these mechanisms and drivers reveal AMR as a complex challenge that needs coordinated efforts from various sectors based on a One Health approach.

Beyond microbial and genetic mechanisms, AMR is influenced by economic, regulatory, and governance factors. Weak enforcement of antimicrobial regulations, fragmented health systems, and limited access to affordable diagnostics promote empiric and inappropriate antimicrobial use. This shows that AMR is not just a microbiological issue; it is a complex socio‐political challenge [45].

## 8 Surveillance and Monitoring Using a One Health Approach

**FIGURE 2:**
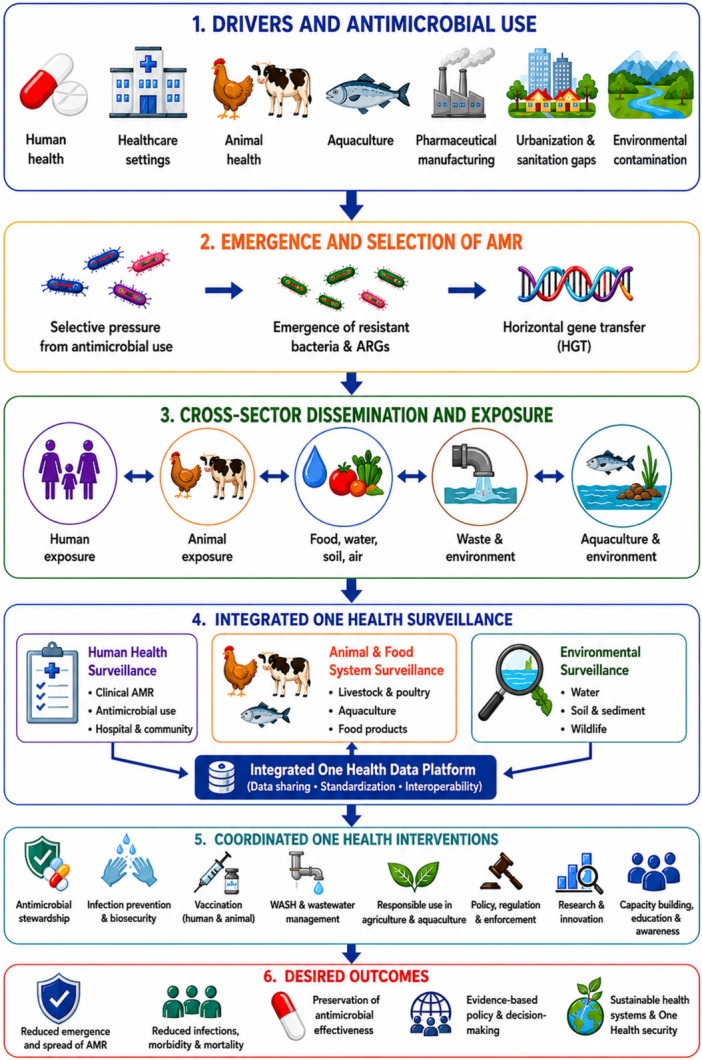
Integrated One Health framework linking antimicrobial use, AMR emergence, cross‐sector surveillance, and coordinated intervention. The framework illustrates the major drivers of AMR, resistance emergence, cross‐sector dissemination, integrated surveillance, data sharing, coordinated interventions, and desired outcomes for AMR control.

Effective surveillance and monitoring are essential for controlling AMR by detecting emerging resistance, tracking trends, and evaluating interventions. A One Health approach to AMR surveillance acknowledges the connections between human, animal, and environmental sectors. It promotes integrated systems that gather resistance data across these areas. This integration is crucial to understand how resistance spreads, identify common causes, and guide coordinated public health efforts (Figure 2).

Integrated AMR surveillance operates globally and nationally (Table 3), with WHO GLASS monitoring human resistance and antimicrobial use, while FAO and WOAH support surveillance in animals and food systems. Environmental monitoring of wastewater, surface water, and soil increasingly complements clinical surveillance [47].

**TABLE 3:** Surveillance systems, policy frameworks, and implementation gaps for antimicrobial resistance under a One Health approach.

Sector	Key surveillance systems/initiatives	Policy frameworks and governance	Major implementation gaps	References
Human health	WHO Global Antimicrobial Resistance and Use Surveillance System (GLASS); national laboratory‐based AMR surveillance networks	WHO Global Action Plan on AMR; national AMR action plans; antimicrobial stewardship policies	Limited laboratory capacity in LMICs; incomplete pathogen coverage; weak linkage between surveillance and clinical decision‐making; underreporting	[1, 2, 9, 46]
Animal health & food production	FAO and WOAH AMR AMU monitoring systems; farm‐level surveillance; food safety monitoring programs	FAO–WOAH–WHO Tripartite AMR framework; guidelines on responsible antimicrobial use in animals	Sparse routine surveillance; lack of standardized indicators; poor regulation of veterinary antimicrobial sales; limited farm‐level compliance	[11, 47, 48]
Environmental sector	Wastewater‐based AMR surveillance; monitoring of surface water, soil, and sediments; research‐based resistome studies	Emerging environmental AMR guidance under One Health; wastewater and pharmaceutical discharge regulations (often fragmented)	Absence of standardized methods; limited integration with human and animal surveillance; weak enforcement of environmental regulations	[6, 25, 28]
Cross‐sector (One Health)	Integrated pilot surveillance platforms; joint risk assessments; multisectoral research initiatives	WHO–FAO–WOAH–UNEP Quadripartite One Health Joint Plan of Action; national One Health coordination mechanisms	Institutional silos; lack of interoperable data systems; legal and ethical barriers to data sharing; insufficient funding and political commitment	[2, 4, 45, 47]
Low‐ and middle‐income countries (LMICs)	Sentinel surveillance sites; donor‐supported laboratory networks; academic‐led initiatives	National AMR action plans aligned with Global Action Plan	Resource constraints; uneven geographic coverage; reliance on external funding; limited workforce training and sustainability	[1, 10, 45]

Abbreviations: AMR, antimicrobial resistance; AMU, antimicrobial use; FAO, Food and Agriculture Organization of the United Nations; GLASS, Global Antimicrobial Resistance and Use Surveillance System; LMICs, low‐ and middle‐income countries; UNEP, United Nations Environment Programme; WHO, World Health Organization; WOAH, World Organisation for Animal Health.

In the animal and environmental sectors, surveillance systems are often scattered or nonexistent, creating major blind spots at the human–animal–environment interface [1]. Additionally, differences in sampling strategies, lab methods, and reporting standards make it hard to compare data across sectors and countries.

Data harmonization is still difficult because human, veterinary, and environmental surveillance systems often work separately and do not connect well. Standardized protocols, shared indicators, interoperable platforms, and data‐sharing frameworks are needed to support integrated cross‐sector AMR surveillance [2, 46].

LMICs face additional barriers, including limited laboratory infrastructure, trained personnel, funding, and access to diagnostics, which hinder integrated One Health surveillance and intervention. Addressing these gaps requires standardized protocols, interoperable data systems, cross‐sector collaboration, capacity building, and sustained political commitment.

## 9 Public Health Interventions and Policy Responses

### 9.1 Antimicrobial Stewardship Across Sectors

Antimicrobial stewardship (AMS) seeks to improve antimicrobial use to decrease resistance while ensuring effective treatment for infections. In human healthcare, AMS programs encourage proper prescribing, following guidelines, optimizing doses, and using de‐escalation strategies. Research shows that strong stewardship efforts can significantly lower the number of multidrug‐resistant organisms, such as MRSA and ESBL‐producing Enterobacterales [49]. In veterinary and food production, stewardship emphasizes limiting the preventive and growth‐promoting use of antimicrobials, implementing treatment guidelines, and encouraging alternatives like vaccination and biosecurity [48]. Coordination across sectors ensures that human, animal, and environmental sources of resistance are addressed together, which reduces the risk of spreading resistance.

### 9.2 Infection Prevention and Control

IPC measures are vital for limiting the spread of resistant pathogens in healthcare, community, and agricultural settings. Key IPC strategies include hand hygiene, proper use of personal protective equipment, cleaning environments, sterilizing medical devices, and isolating infected patients. In livestock and aquaculture, biosecurity actions—such as limited access, good waste management, and disinfection methods—help reduce pathogen transmission among animals and from animals to humans [45, 50]. Improving IPC not only lowers infection rates but also cuts unnecessary antimicrobial use, which helps lessen the pressure that contributes to resistance.

Vaccination is an important but often overlooked way to reduce AMR. By decreasing the number of bacterial and viral infections, vaccines help lower the use of antimicrobials and the resulting pressure for resistance. Increasing the use of vaccines in humans and animals can greatly support AMR control within a One Health framework [51, 52].

### 9.3 Regulatory Frameworks and Global Initiatives

Global efforts through regulatory frameworks and initiatives support sustainable AMR management. The WHO Global Action Plan on AMR provides goals, including enhancing surveillance, reducing infection rates, and optimizing antimicrobial use. The FAO and WOAH focus on monitoring antimicrobial use in animals, setting standards for responsible practices, and promoting collaboration through One Health [47]. National policies, like regulations for prescription‐only antibiotics, audits of antimicrobial use, and standards for environmental discharge, support these global aims. Working together on these frameworks ensures consistent approaches across human, animal, and environmental sectors, treating AMR as a worldwide public health concern.

Overall, coordinated actions that combine antimicrobial stewardship, IPC, and strong regulatory frameworks are crucial for fighting AMR. The One Health approach promotes collaboration across sectors, evidence‐based policies, and sustainable practices, allowing countries to lessen the public health impact of AMR while safeguarding the effectiveness of antimicrobials for future generations.

## 10 Challenges and Knowledge Gaps

Despite progress in understanding AMR, methodological inconsistencies, limited environmental monitoring, and lacking diagnostic capacity hold back effective surveillance and policy implementation. Variations in sampling, lab procedures, and reporting make it hard to integrate data across different sectors. Limited guidelines for environmental monitoring and insufficient infrastructure, along with a lack of trained personnel in LMICs, lead to incomplete datasets and unclear risk assessment [1, 20].

Implementing One Health interventions faces several barriers. Institutional silos, broken governance structures, and poor coordination between sectors hinder the application of One Health strategies. Human, veterinary, and environmental agencies often work independently and share little surveillance data or collaborate on interventions [47]. Even where policies are in place, enforcement can be inconsistent, and there are often few incentives for compliance. These systemic challenges weaken antimicrobial stewardship efforts, infection prevention and control measures, and regulations meant to reduce AMR.

Equity and resource limitations disproportionately burden LMICs, which face high infectious disease rates, antimicrobial use, and environmental contamination but often lack the financial, technical, and human resources for comprehensive One Health strategies. Disparities between urban and rural areas, along with socioeconomic inequalities, make stewardship and surveillance even more difficult. This highlights the urgent need for targeted investments in capacity building, technology transfer, and infrastructure [45].

Together, controlling AMR is limited by methodological, institutional, and resource challenges. Closing these knowledge and implementation gaps requires coordinated efforts to standardize surveillance, improve cross‐sector collaboration, and allocate resources fairly. Only through such inclusive approaches can One Health strategies be effectively put into practice to lessen the global impact of AMR.

Furthermore, ethical and equity issues are major challenges in controlling AMR. Balancing the responsible use of antimicrobials with fair access to essential medicines is especially difficult in LMICs. Restrictive policies can unintentionally reduce treatment options. To tackle AMR, governance frameworks are needed that support safe use while ensuring access and health equity.

## 11 Future Directions and Research Priorities

### 11.1 Strengthening Interdisciplinary Collaboration

Interdisciplinary collaboration is key to closing the gaps between human health, veterinary medicine, agriculture, and environmental science. Multi‐sector research teams can promote knowledge sharing, standardize methods, and align data collection across different areas. These collaborative efforts should involve clinicians, veterinarians, microbiologists, ecologists, policymakers, and social scientists to ensure that interventions are specific to their context, sustainable, and effective [45]. Involving communities and stakeholders at local and regional levels is also crucial to encourage behavioral change and ensure adherence to antimicrobial stewardship and infection prevention measures.

### 11.2 Innovations in Surveillance and Diagnostics

Advances in metagenomics, whole‐genome sequencing, CRISPR‐based diagnostics, bioinformatics, and artificial intelligence allow for sensitive detection and tracking of AMR across human, animal, and environmental sectors [4, 6]. Combining genomic data with environmental monitoring and real‐time digital surveillance can improve predictive modeling, early‐warning systems, and targeted interventions.

### 11.3 Policy Priorities and Implementation Strategies for AMR Control

Effective AMR control requires coordinated One Health policies integrating human, animal, aquaculture, and environmental health sectors. Key priorities include multisectoral governance, integrated surveillance and data sharing, strengthened diagnostic and laboratory capacity, antimicrobial stewardship, environmental protection, and workforce development. In LMICs, interventions should be affordable, context‐specific, and adapted to local health‐system capacity. Priority actions include strengthening existing laboratories and surveillance networks, improving access to quality‐assured diagnostics and antimicrobials, promoting vaccination, biosecurity, and improved animal husbandry, and managing wastewater and pharmaceutical waste. Sustainable financing, community engagement, regional collaboration, interdisciplinary research, and effective governance are essential for translating national AMR action plans into measurable outcomes, protecting public health, ensuring equity, and preserving antimicrobial effectiveness for future generations.

## 12 Conclusions

Antimicrobial resistance is a complex and interconnected One Health challenge shaped by antimicrobial use, healthcare practices, food production, environmental pollution, human movement, and ecological interactions. Resistance does not remain within individual sectors; resistant organisms and antimicrobial resistance genes can circulate between humans, animals, food systems, water, soil, wastewater, and wildlife through various interconnected pathways. The environment is therefore not just a passive recipient of antimicrobial residues and resistant organisms but also an important source and route for spreading resistance.

Effective AMR control requires coordinated action across different sectors rather than isolated interventions. Priority measures include responsible antimicrobial use, strengthened antimicrobial management, improved infection prevention and control, expanded diagnostic capacity, integrated human–animal–environment surveillance, environmental monitoring, unified data systems, and effective regulation of antimicrobial use and waste. Particular attention is needed in resource‐limited settings, where surveillance and implementation capacity remain uneven. Sustained political commitment, interdisciplinary collaboration, equitable financing, and evidence‐based policy are essential to translate the One Health concept into effective and lasting AMR prevention and control.

## Author Contributions

**Nurshad Ali:** conceptualization, writing – review and editing, writing – original draft.

## Funding

The author has nothing to report.

## Disclosure

The lead/corresponding author (Nurshad Ali) affirms that this manuscript is an honest, accurate, and transparent account of the study being reported; that no important aspects of the study have been omitted; and that any discrepancies from the study as planned (and, if relevant, registered) have been explained.

## Ethics Statement

The author has nothing to report.

## Conflicts of Interest

The author declares no conflicts of interest.

## Data Availability

Data sharing is not applicable to this article, as no new data were created or analyzed in this study.
